# Novel Ascorbic Acid Co-Crystal Formulations for Improved Stability

**DOI:** 10.3390/molecules27227998

**Published:** 2022-11-18

**Authors:** Hui Zhang, Huahui Zeng, Mengfei Li, Yagang Song, Shuo Tian, Jing Xiong, Lan He, Yang Liu, Xiangxiang Wu

**Affiliations:** 1 Pharmacy College, Henan University of Chinese Medicine, Zhengzhou 450046, China; 2 Academy of Chinese Medicine Sciences, Henan University of Chinese Medicine, Zhengzhou 450046, China; 3 National Institutes for Food and Drug Control, Beijing 102629, China

**Keywords:** co-crystals, ascorbic acid, improved stability, hydrogen bonding

## Abstract

A series of co-crystals of ascorbic acid were prepared with equimolar amounts of co-crystal formers (CCFs), including isonicotinic acid, nicotinic acid, 3,4-dihydroxybenzoic acid, 2,5-dihydroxybenzoic acid and m-hydroxybenzoic acid, by slow solvent evaporation and solvent-assisted grinding. The co-crystals were characterized by single-crystal X-ray diffraction spectroscopy, powder X-ray diffraction, IR spectroscopy, differential scanning calorimetry and thermogravimetric analysis. Molecular dynamics (MD) simulations further validated the interaction energy and the possible intermolecular hydrogen bonds among VC and CCFs. The co-crystals showed improved stability when exposed to different wavelengths of light, pH and temperatures compared to the free analogue, especially at higher pH (~9) and lower temperature (~4 °C).

## 1. Introduction

The key process in drug development is pre-formulation [1], which is significantly influenced by the physicochemical properties of the drugs [2]. To improve the physicochemical and pharmacokinetic properties of drugs, they are prepared in different solid forms, such as polycrystalline [3], amorphous [4], salts, hydrates or solvates [5], and co-crystals [6]. However, most solid forms of drugs have limitations; for example, only ionic fractions of drugs are suitable for salt formation, whereas hydrates or solvates are often unstable or toxic [7,8], and water or solvent molecules dissipate in a time-dependent manner [2]. In recent years, drug co-crystals have attracted considerable attention in drug design [9]. Drug co-crystals are formed by active drug ingredients (APIs) and co-crystal formers (CCFs) in the same lattice according to certain stoichiometric ratios and are bound together by non-covalent bonds, such as hydrogen bonding, π-π stacking and Van der Waals interactions. The co-crystallization of drugs improve their physicochemical properties, such as their melting point, solubility [10], stability [11], hygroscopicity, bioavailability [12], permeability [6], and intrinsic solubility [13]. It is necessary to evaluate the binding modes between API and CCFs in order to select suitable CCFs for achieving predictable drug structures and properties. While single-crystal diffraction provides the most direct evidence of binding modes, molecular dynamics (MD) predicts atom positions and velocities by taking small time increments based on Newton’s equation of motion [14]. MD simulations can help understand the molecular interactions between API and CCFs or between co-crystals and selected solvates or excipients [15]. MD simulations have been extensively used to study the solubility and miscibility of APIs in the presence of CCFs or excipients [16].

Ascorbic acid, known as vitamin C (VC), is a water-soluble vitamin that was first synthesized by the Swiss chemist Tadeus Reichstein in 1933. It is used to treat anemia and sepsis [17] and is used as a dietary supplement to prevent atherosclerosis and inflammation. In addition, topical ascorbic acid formulations are used for skin whitening and removing melanin deposits. However, since the ascorbic acid molecule contains two chiral carbon atoms, it is prone to tautomerism in aqueous solutions. Furthermore, the alkene diol structure of ascorbic acid has strong reducibility, and its lactone ring is easily hydrolyzed, which hinders its clinical applications. Although the encapsulation of ascorbic acid into liposomes, nanoparticles and microcapsules can improve its stability, its low encapsulation rate and drug loading capacity are major deterrents. The aim of this study was to prepare ascorbic acid co-crystals with enhanced stability and malleability.

**Scheme 1:**
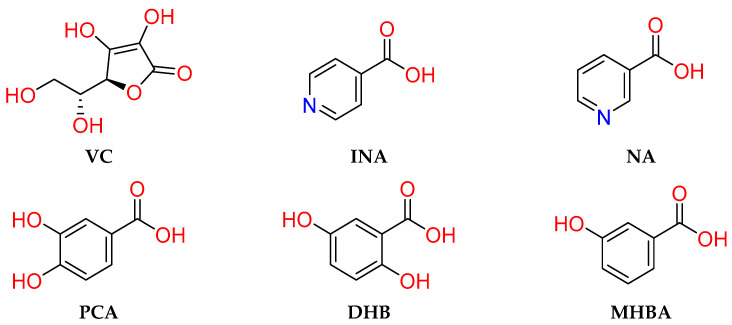
Ascorbic acid (VC) and coformers in the present study.

The two hydroxyl groups of ascorbic acid can act as both donors and acceptors for hydrogen bonds and may form hydrogen bonds with CCFs bearing carboxyl and hydroxyl groups. Although several ascorbic acid co-crystals have been reported in recent years, a systematic correlation has not been established between the co-crystal structures and the mechanical properties [18]. The co-crystals of VC with a series of CCFs containing carboxyl and hydroxyl groups, such as isonicotinoid, nicotinamide, nicotinic acid, 4,4-bispyridine, arginine, serine, and 2-amino-6-methylpyridine, have been reported before [19,20,21,22,23,24,25,26]. As part of an ongoing study in crystal engineering, we examined co-crystals of VC with similar CCFs, including isonicotinic acid (INA), nicotinic acid (NA), 3,4-dihydroxybenzoic acid (PCA), 2,5-dihydroxybenzoic acid (DHB) and m-hydroxybenzoic acid (MHBA) (Scheme 1), which are all in the GRAS list of FDA [27]. In addition, the ΔpKa values of the CCFs are in the range of 0.19~1.28, which negates any possibility of proton transfer [28,29] and indicates the possibility of co-crystal formation. It was a pity only the single crystal of VC-INA (1:1) was characterized by SC-XRD. However, extensive MD simulations of all the co-crystals by Gromacs were performed to mimic the experimental scenario by constructing the simulation models in the presence of a dissolution medium water. The models were prepared in order to monitor the hydrogen bonding and the interaction energy between VC and CCFs and predict the binding mode and stability. Powder X-ray diffraction (PXRD), Fourier transform infrared (FT-IR), differential scanning calorimetry (DSC) and thermogravimetric analysis (TG) were also performed to confirm the formation of co-crystals and assess the binding modes. The stability of the eutectics under different light, temperature and pH conditions was also investigated.

## 2. Results and Discussion

### 2.1. Crystal Structure Analysis

The single crystal structure was analyzed by the Shelxtl software package v6.1 (University of Göttingen, Göttingen, Germany). The crystallographic data of the VC-INA co-crystal is summarized in Table 1. Selected bond lengths (Å) and bond angles (°) were listed in Table S1 of Supplementary Materials.

**Scheme 2:**
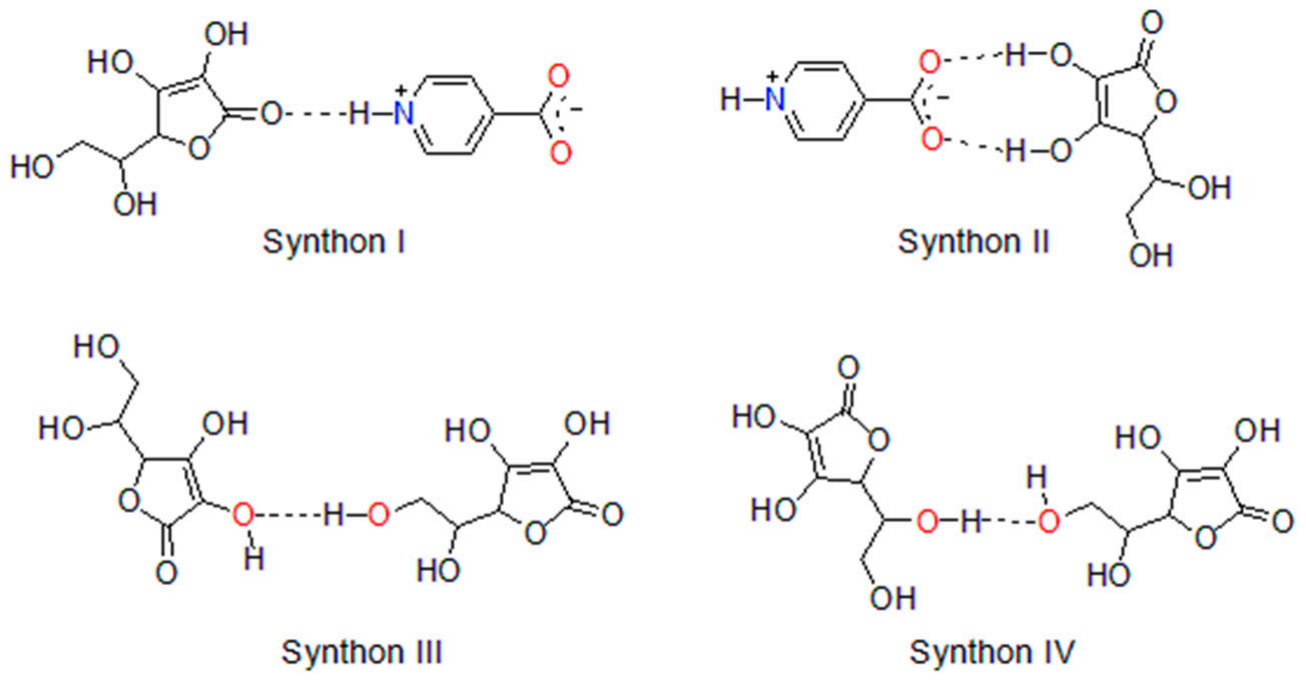
Common synthons observed in the co-crystals.

The supramolecular synthons joined by hydrogen bonds are shown in Scheme 2. The electron-density difference maps showed that the H atom was transferred from the –COOH group to the N atom. The difference between C–O and C=O distances for the unprotonated and protonated –COOH groups also indicated the formation of hydrogen bonds. In the VC-INA co-crystal, the C–O and C=O bonds differed by 0.05 Å compared to 0.08 Å in the protonated –COOH group.

**Figure 1:**
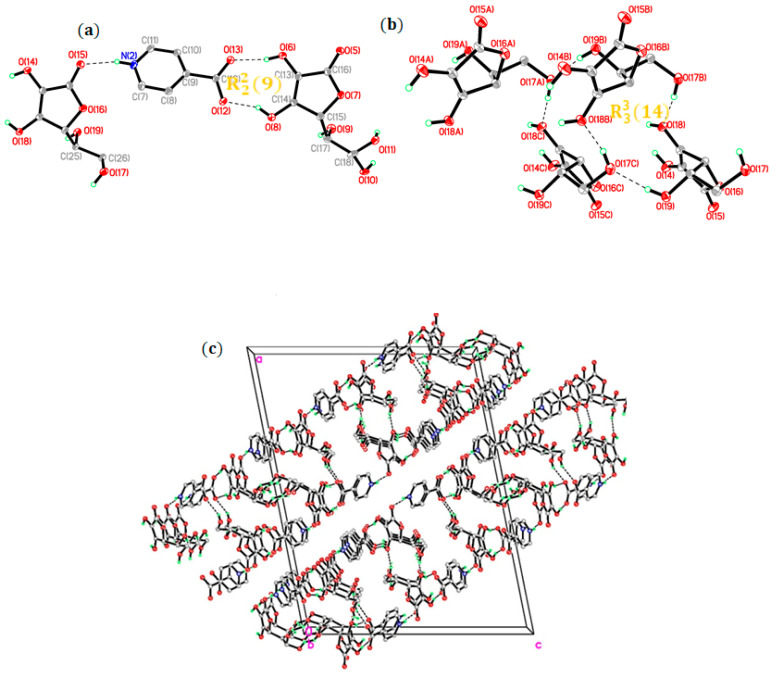
Hydrogen-bonded pattern among (**a**) Type I, (**b**) Type II and (**c**) 2D ribbon in VC-INA.

Two different intermolecular hydrogen bonds were detected in the crystal structure—one between VC and INA and the other between VC itself. The VC and INA molecules were combined via synthons I and II, wherein the two enol hydroxyl groups of VC and the —COO^−^ fragment of the zwitterion of INA were linked via two O—H···O hydrogen bonds to form an $R_{2}^{2}\left(9\right)$ ring motif (Figure 1a), similar to that reported for the co-crystal of ascorbic acid and pyridine-2-carboxylic acid [18]. The N—H in INA acted as the hydrogen bond donor, and the —C=O of VC acted as the acceptor in an infinite one-dimensional (1D) chain. The adjacent 1D chains were joined through synthons III and IV between VC molecules, which generated a closed-quadrangle cavity measuring 6.0 × 6.5 Å^2^ arranged in a $R_{3}^{3}\left(14\right)$ ring motif (Figure 1b) that formed a two-dimensional (2D) ribbon surface along the (20$\bar{1}$) direction (Figure 1c).

### 2.2. MD Simulations

In general, a decrease in binding energy indicates a favorable interaction. The strength of the molecular interactions was quantified by calculating the electrostatic (*E*_coul_), Lenard–Jones (*E*_LJ_) and total energies (*E*_total_). The total energy of the co-crystal systems was lower compared to the sum of the monomers, indicating favorable interactions in all co-crystals (Table 2). Furthermore, the total energy was the lowest for VC-INA, followed by VC-NA, VC–MHBA, VC-PCA and VC-DHB in ascending order. This trend was consistent with the average number of hydrogen bonds between VC and the different CCFs (Table 3), indicating that intermolecular hydrogen bonds are responsible for stabilizing the co-crystal system.

**Figure 2:**
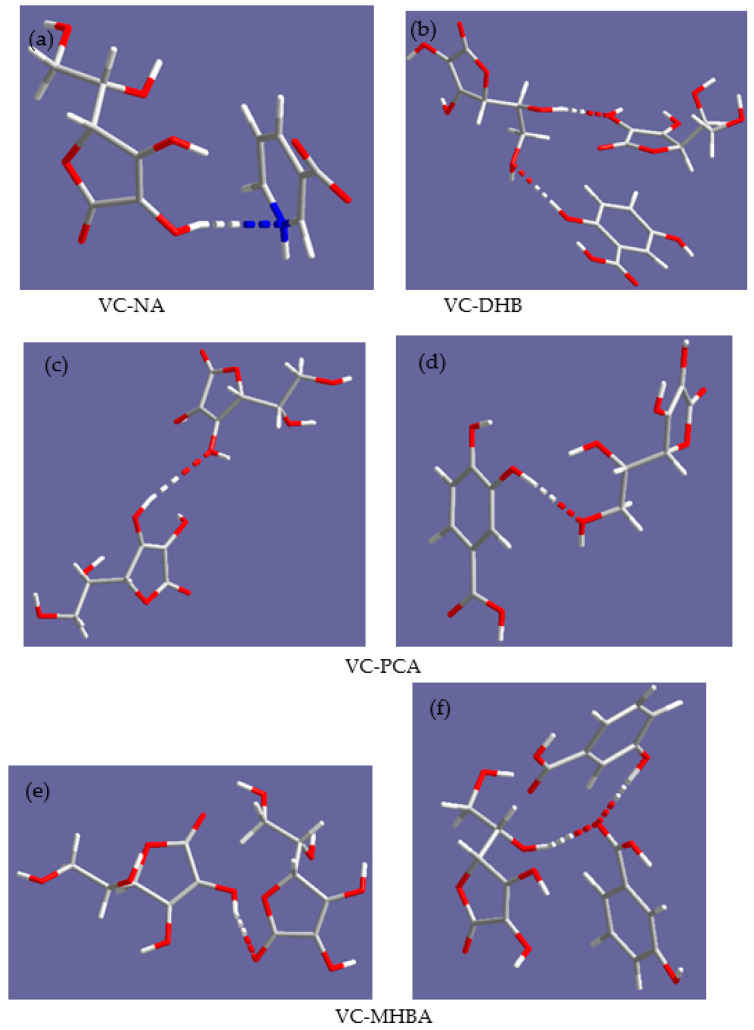
(**a**): VC-NA, (**b**): VC-DHB, (**c**,**d**): VC-PCA, (**e**,**f**): VC-MHBA. Probable interactions between VC and CCFs. All the molecules are represented with wires and sticks; C, H, O and N are represented by gray, white, red and blue, respectively.

Although the MD simulations could not fully reflect the accurate interaction patterns, the trajectory analysis showed that hydrogen bonds are important driving forces in the formation of new co-crystals. In addition, we observed different hydrogen bonding patterns during the simulation. The enol hydroxyl groups of VC formed hydrogen bonds between VC itself in the VC-DHB, VC-PCA and VC-MHBA co-crystals and between VC and NA in the VC-NA co-crystal (Figure 2).

### 2.3. Thermal Analysis

**Figure 3:**
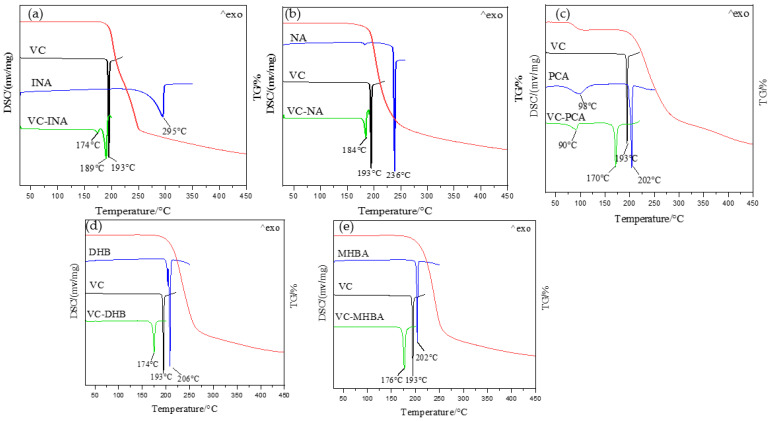
Schematic diagram of thermal analysis ((**a**): DSC and TG images of VC-INA co-crystal; (**b**): DSC and TG images of VC-PCA co-crystal; (**c**): DSC and TG images of VC-NA co-crystal; (**d**): DSC and TG images of VC-DHB co-crystal; (**e**): DSC and TG images of the VC-MHBA co-crystal).

The thermal behavior of the co-crystals was determined by TG and DSC, and ascorbic acid and CCFs were used as the reference. As shown in Figure 3, ascorbic acid and the CCFs had different endothermic peak positions compared to the co-crystals, which was indicative of distinct thermal behavior. The TG and DSC curves showed that all the co-crystals except for VC-PCA are free from crystalline water or solvents in the lattice and began to decompose at approximately 189 °C, 184 °C, 174 °C and 176 °C for VC-INA, VC-NA, VC-DHB, VC-MHBA, respectively (Figure 3). The values are all lower than VC or CCFs, and the phenomenon is consistent with the majority of co-crystals. The DSC curve of the VC-PCA co-crystal showed an endothermic peak at 90 °C, indicating the volatilization of the crystal water or solvent. Accordingly, the results of TG thermograms showed VC-PCA first underwent a weight loss of 4.6%, then began to decompose at 170 °C with a weight loss of 51%.

### 2.4. X-ray Powder Diffraction

**Figure 4:**
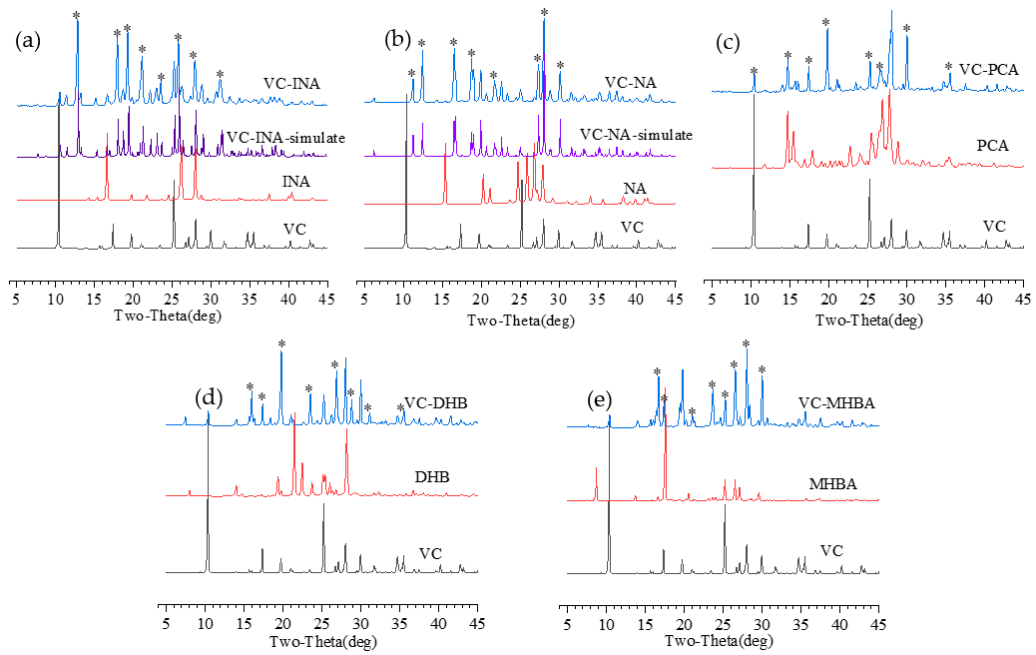
Schematic diagram of PXRD patterns of (**a**): VC-INA; (**b**): VC-PCA; (**c**): VC-NA; (**d**): VC-DHB; (**e**): VC-MHBA. * represents the characteristic diffraction peaks of co-crystals.

The PXRD spectra of all the monomers and co-crystals are shown in Figure 4. The VC-INA curves showed significant differences compared to VCs and CCFs. The newly formed peaks of the VC-INA co-crystals appeared at 12.82°, 19.28°, 17.94°, 25.8°, 21.12°, 27.92°, 31.14° and 23.54°. Moreover, the experimental PXRD pattern from grinding was consistent with the simulated pattern calculated from the structural parameters of the present single crystal of VC-INA or with the reported one for VC-NA [19,20], confirming the crystalline phase purity of the grinding co-crystal (Figure 4a,b). Although other single-crystal attempts had failed, a similar PXRD pattern variation was observed in other co-crystals. All the co-crystals showed characteristic diffraction peaks that differed from VC and CCFs, suggesting the formation of a new crystalline phase. The purity can be proven by the disappearance of characteristic diffraction peaks of their parent materials.

### 2.5. Fourier Transform Infrared Analysis

**Figure 5:**
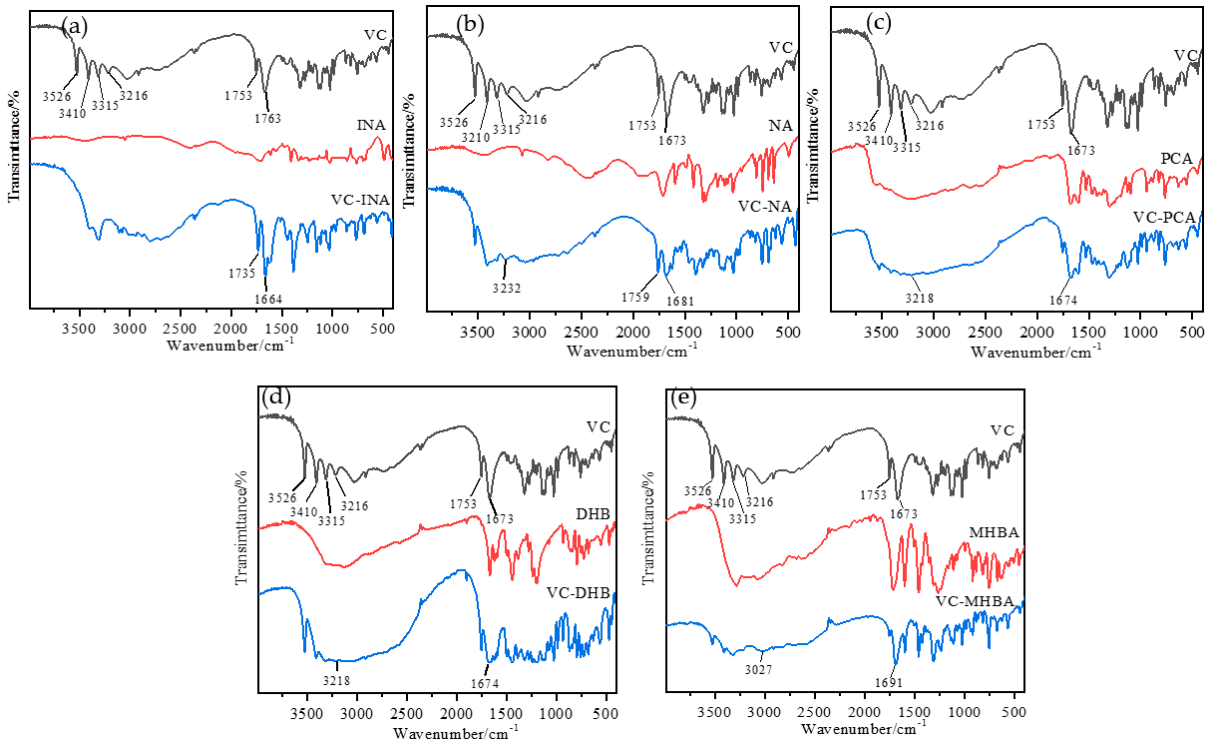
FT-IR analysis plots of (**a**): VC-INA; (**b**): VC-PCA; (**c**): VC-NA; (**d**): VC-DHB; (**e**): VC-MHBA.

IR spectroscopy can identify characteristic functional groups and their intermolecular interactions, which are manifested by a shift in the vibrational frequency [30]. The IR spectra of VC, INA and VC-INA co-crystals are shown in Figure 5. The IR spectrum of VC showed characteristic peaks at 1753 cm^−1^ and 1673 cm^−1^ corresponding to the stretching of the carbonyl (C==O) group, and quadruple characteristic peaks at 3526 cm^−1^, 3410 cm^−1^, 3315 cm^−1^ and 3216 cm^−1^ for the stretching of the hydroxyl (O—H) group [31]. The IR spectrum of the VC-INA co-crystal was distinct from that of VC or INA. The characteristic peak of VC shifted to 1735 cm^−1^ and 1664 cm^−1^ in VC-INA, which was attributed to the formation of the H-bond from the carbonyl group. The IR spectrum of the VC-PCA co-crystal showed the shift of characteristic peaks to 3218 cm^−1^ and 1674 cm^−1^. Furthermore, the presence of O—H stretching vibrations in the VC-NA co-crystal was indicated by the peak at 3232 cm^−1^. The characteristic peak of VC also shifted to 1759 cm^−1^ and 1681 cm^−1^. The infrared spectrum of the VC-DHB co-crystal showed characteristic peak shifts to 3412 cm^−1^ and 3318 cm^−1^ and the shift of the carbonyl group (C==O) to 1671 cm^−1^. For the VC-MHBA, the characteristic peaks shifted to 3027 cm^−1^ and 1691 cm^−1^, which was indicative of the existence of hydrogen bonds.

### 2.6. Stability Analysis

**Figure 6:**
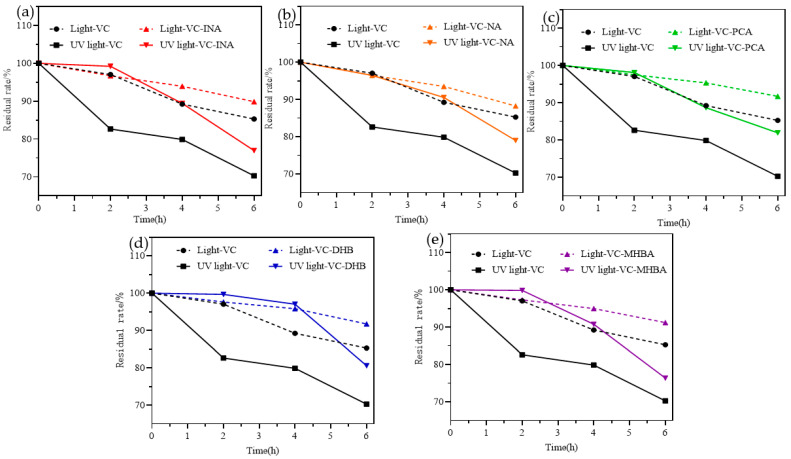
Photostability of VC co-crystals ((**a**): VC-INA; (**b**): VC-PCA; (**c**): VC-NA; (**d**): VC-DHB; (**e**): VC-MHBA).

**Figure 7:**
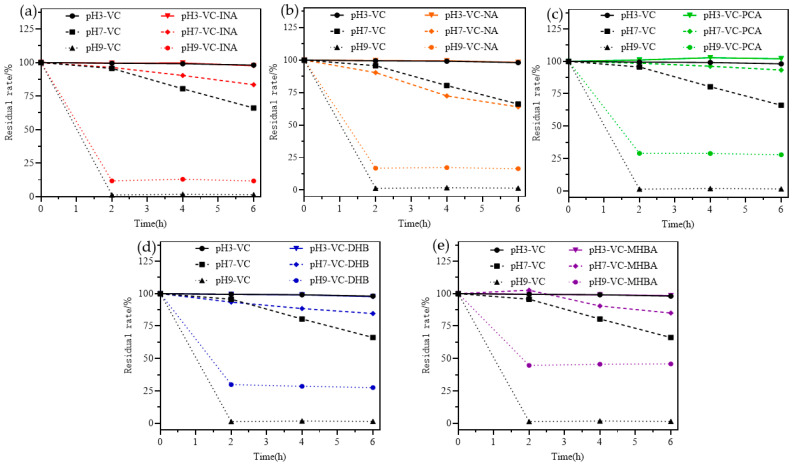
pH stability experiment of VC co-crystals ((**a**): VC-INA; (**b**): VC-PCA; (**c**): VC-NA; (**d**): VC-DHB; (**e**): VC-MHBA).

**Figure 8:**
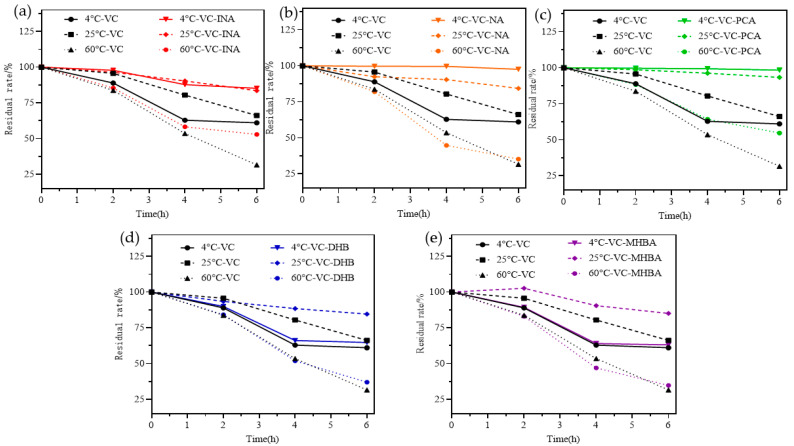
Thermostability of VC co-crystals ((**a**): VC-INA; (**b**): VC-PCA; (**c**): VC-NA; (**d**): VC-DHB; (**e**): VC-MHBA).

As shown in Figure 6, both sunlight and UV light exposure decreased the residual amount of VC in a time-dependent manner, and the latter had a stronger effect. This is consistent with the fact that VC undergoes oxidation when exposed to light. Furthermore, the residual amounts of VC-INA, VC-PCA, VC-NA, VC-DHB and VC-MHBA co-crystals were higher compared to that of VC after 6 h. The effect of pH on VC and its co-crystals is shown in Figure 7. The residual amounts of VC and the co-crystals were almost 98% under acidic conditions, and the neutral pH did not have a significant effect, although the stability of all VC co-crystals was higher than that of VC. At the alkaline pH of ~9, the residual amount of both VC and VC co-crystals decreased sharply to less than 50% after 2 h, and VC was more susceptible compared to the co-crystals. As shown in Figure 8, 31% of the VC co-crystals remained at 60 °C, which was still higher than that of VC. Taken together, these results suggested that co-crystal formation can improve the photo-, pH and thermal stability of VC, especially at higher pH (~9) and lower temperature (~4 °C).

## 3. Materials and Methods

### 3.1. Materials

Ascorbic acid, isonicotinic acid, 3,4-dihydroxybenzoic acid, 2,5-dihydroxybenzoic acid, niacin acid and m-hydroxybenzoic acid were purchased from Shanghai Aladdin Biochemical Technology Co. Ltd. (Shanghai, China). Ethanol (chromatographically pure) was purchased from Aladdin Reagents, Inc. (Shanghai, China). All other reagents were of analytical grade and commercially available.

### 3.2. Dynamic Simulation

Scheme 1 represents the planar view of the molecular structure of VC and the CCFs. The GROMACS v2019.6 package (KTH-Royal Institute of Technology, Stockholm, Sweden) was used to perform all the MD simulations [32]. The systems were modeled using the General AMBER Force Field (GAFF) force field, a general-purpose force field with wide applicability, especially for small molecules [33]. The parameter files were generated using the sobtop_1.0(dev3.1) tool.

The SPC/E model was used for water molecules [34]. Based on the formation mechanism and the crystal structure of VC-INA, the model of each VC-CCF co-crystal was constructed by randomly inserting the CCF molecules into VC molecules at the molar ratio of 1:1. The initial model of VC-CCF consisted of 15 VC and an equivalent amount of CCF molecules. All initial models were kept in the center of a large simulation box measuring ∼5 nm × 5 nm × 5 nm and solvated with water. Using the steepest descent method, all the co-crystal systems were initially subjected to energy minimization. The solvated systems were simulated at room temperature using an isothermal and isochoric MD scheme, followed by isobaric-isothermal MD simulations at 1 bar. The equations of motion were integrated by the leapfrog algorithm with 2-fs time steps, and periodic boundary conditions were applied in all directions.

### 3.3. Co-Crystal Preparation

The VC co-crystals were prepared by solvent-assisted grinding. Briefly, equimolar amounts of VC and the CCF were placed in a 2 mL grinding tube containing grinding beads, and an appropriate amount of ethanol or pure water was added as a solvent. The mixture was placed in the stainless-steel jar of the grinder and vibrated at 53 Hz at room temperature. After 20 min, the co-crystals were placed in a vacuum desiccator for 4 h at 55 °C.

### 3.4. Single-Crystal X-ray Diffraction Analysis

Single crystals of VC-INA (C_12_H_13_NO_8_) were obtained from the solvent EtOH/PhMe (1:1, *v*/*v*), and a suitable crystal was selected for X-ray diffraction (SC-XRD) on a Super Nova, Dual, Cu at zero, AtlasS2 diffractometer (Agilent Technologies, Santa Clara, CA, USA). The crystal was kept at 150.00(10) K during data collection.

### 3.5. Differential Scanning Calorimetry

The thermal behavior of the co-crystal was evaluated by differential scanning calorimetry (DSC, Mettler Toledo DSC3; Mettler Toledo, Columbus, OH, USA). Briefly, 2–3 mg of the co-crystal was placed in an alumina crucible covered with holes and then scanned at the rate of 10 °C/min with 20 mL/min nitrogen in the range of 30 °C to 350 °C.

### 3.6. X-ray Powder Diffraction

PXRD data were collected using a Rigaku Smart Lab 9 kW powder X-ray diffractometer (Tokyo, Japan). Briefly, 50 mg of the co-crystal was loaded into a flat quartz glass sample slide with etched squares and subjected to Cu-Kα radiation at an acceleration voltage of 45 kV and with a 200 mA current. The samples were scanned in the continuous mode in the 2θ range from 5° to 60° at a scan rate of 10° min^−1^ and 0.02° scan steps.

### 3.7. FT-IR Microscopy System

The variation in the characteristic peaks was observed using an FT-IR microscope (Bruker INVENIO; Billerica, MA, USA) with KBr compression. After subtracting the background blank, data were acquired at wavelengths from 4000 to 400 cm^−1^, and the IR spectra were established after 16 scans of each spectrum.

### 3.8. Photostability Analysis

Briefly, 30 mg ascorbic acid and its co-crystals were weighed separately and dissolved in 15 mL distilled water. The samples were placed under daylight and UV (wavelength 253.7 nm) for 2, 4 and 6 h, and the absorbance was measured at 245 nm to calculate the residual levels of the substance.

### 3.9. Thermostability Analysis

The samples were prepared as above and incubated at 4 °C, 25 °C and 60 °C in the dark for 2, 4 and 6 h. The absorbance of the solutions was measured at 245 nm.

### 3.10. pH Stability Analysis

The co-crystal samples were prepared as described, and 0.1% HCl or 0.1% NaOH was added to adjust the pH of the solutions to 3, 7 and 9. The samples were incubated in the dark for 2, 4 and 6 h, and the absorbance was measured at 245 nm.

## 4. Conclusions

We successfully prepared co-crystals of ascorbic acid with different co-crystal formers (CCF) at the molar ratio of 1:1 by slow solvent evaporation and solvent-assisted grinding and verified our findings by molecular dynamics (MD) simulations and experimental approaches. Co-crystal formation improved the stability of VC in comparison with the monomer form, especially at higher pH (~9) and lower temperature (~4 °C), which may be related to the abundant hydrogen bonds between the VC and CCF molecules. The hydrogen bonds formed by the enol hydroxyl groups, in particular, stabilized the lactone ring of VC. Additionally, the decrease in binding energy also indicates a more favorable interaction. Our study provided insights into the relationship between the co-crystal structure and properties and provides an experimental basis for drug crystal engineering strategies.

## Figures and Tables

**Table 1:** Crystallographic data of the VC-INA co-crystal.

Compound	VC-INA
Empirical formula	C_12_H_13_NO_8_
Formula weight	299.23
Temperature/K	150.00(10)
Crystal system	monoclinic
Space group	C2 (no. 5)
a/Å	30.073(2)
b/Å	5.4222(4)
c/Å	23.6087(12)
α/°	90
β/°	101.250(5)
γ/°	90
Volume/Å^3^	3775.7(4)
Z	12
ρ_calc_g/cm^3^	1.579
μ/mm^−1^	1.173
F(000)	1872.0
Index ranges	−35 ≤ h ≤ 36, −6 ≤ k ≤ 4, −29 ≤ l ≤ 24
Data/restraints/parameters	5980/1/597
Final R indexes (I ≥ 2σ(I))	R_1_ = 0.0830, wR_2_ = 0.2078
Final R indexes (all data)	R_1_ = 0.0975, wR_2_ = 0.2227
Goodness-of-fit on F^2^	1.014
Flack parameter	−0.1(4)

**Table 2:** Interaction energies (kJ·mol^−1^) of VC/co-crystals without water.

System	*E*_coul_	*E*_LJ_	*E*_total_	Δ*E* *
		VC-INA		
VC	−3088.88	−306.384	−3395.26	
INA	−241.67	−222.065	−463.735	
VC-INA	−3369.94	−921.246	−4291.19	−432.195
		VC-NA		
VC	−3088.19	−301.88	−3390.07	
NA	438.305	−237.564	200.741	
VC-NA	−2687.16	−940.937	−3628.1	−438.771
		VC-PCA		
VC	−3093.84	−319.397	−3413.24	
PCA	−2367.44	−215.397	−2582.84	
VC-PCA	−5569.87	−965.21	−6535.08	−539.000
		VC-DHB		
VC	−3098.22	−336.478	−3434.7	
DHB	1124.42	−132.278	992.142	
VC-DHB	−2084.45	−902.098	−2986.55	−543.992
		VC-MHBA		
VC	−3090.57	−322.092	−3412.66	
MHBA	−309.506	−184.255	−493.761	
VC-MHBA	−3481.98	−890.006	−4371.99	−465.569

*: Δ*E*_total_ = *E*_API-CCF_ − (*E*_API_ + *E*_CCF_).

**Table 3:** Number of hydrogen bonds in VC-CCFs without water.

System	H-Bond Number
VC-INA	20.55
VC-NA	22.07
VC-PCA	47.05
VC-DHB	48.05
VC-MHBA	32.33

## Data Availability

All experimental data required to reproduce the findings from this study will be made available to interested investigators.

## Supplementary Materials

The following supporting information can be downloaded at: https://www.mdpi.com/article/10.3390/molecules27227998/s1, Table S1: selected bond lengths (Å) and bond angles (°). Table S2. Hydrogen bond length (Å) and bond angle (°).

## Sample Availability

Samples of the compounds of VC-INA, VC-PCA, VC-NA, VC-DHB and VC-MHBA) are available from the authors.
