# Supplementary material for: Novel Ascorbic Acid Co-Crystal Formulations for Improved Stability
Source: Molecules. 2022 Nov 18;27(22):7998. doi: 10.3390/molecules27227998 (PMC9699323; doi:10.3390/molecules27227998)
Supplement: Supplementary file 1 [file molecules-27-07998-s001.zip › molecules-1996004-supplementary.pdf]

Supporting Information for

Novel Ascorbic Acid Co-Crystal Formulations for Improved Stability

Hui Zhang [1,†], Huahui Zeng [2,†], Mengfei Li [1,2], Yagang Song [2], Shuo Tian [2], Jing Xiong [3], Lan He [3], Yang Liu [3,*] and Xiangxiang Wu [1,2,*]

[1] Pharmacy College, Henan University of Chinese Medicine, Zhengzhou 450046, China
[2] Academy of Chinese Medicine Sciences, Henan University of Chinese Medicine, Zhengzhou 450046, China
[3] National Institutes for Food and Drug Control, Beijing 102629, China
* Correspondence: wuxx-415@126.com (X.W.); yangliu@nifdc.org.cn (Y.L.); Tel.: +86-371-65680206 (X.W.); +86-10-53851571 (Y.L.)
† These authors contributed equally to this work.

Table of Contents

Table S1. selected bond lengths (Å) and bond angles (°).
Table S2. Hydrogen bond length (Å) and bond angle (°).

Table S1. selected bond lengths (Å) and bond angles (°).

C(1)-C(2)	1.515(9)	C(13)-C(14)	1.347(9)
C(2)-C(3)	1.378(9)	C(13)-C(16)	1.450(9)
C(2)-C(6)	1.397(9)	C(14)-C(15)	1.495(9)
C(3)-C(4)	1.384(9)	C(15)-C(17)	1.529(8)
C(5)-C(6)	1.383(9)	C(17)-C(18)	1.503(10)
C(19)-C(20)	1.389(10)	O(12)-C(12)	1.279(8)
C(20)-C(21)	1.382(9)	O(13)-C(12)	1.225(8)
C(21)-C(22)	1.390(9)	N(2)-C(7)	1.338(9)
C(21)-C(24)	1.517(9)	N(2)-C(11)	1.328(9)
C(22)-C(23)	1.387(10)	C(7)-C(8)	1.385(10)
N(1)-C(4)	1.333(8)	C(8)-C(9)	1.379(9)
N(1)-C(5)	1.341(8)	C(9)-C(10)	1.382(9)
N(3)-C(19)	1.344(9)	C(9)-C(12)	1.525(9)
N(3)-C(23)	1.331(9)	C(10)-C(11)	1.386(10)
O(1)-C(24)	1.237(8)	O(14)-C(29)	1.332(8)
O(2)-C(24)	1.260(7)	O(15)-C(30)	1.223(8)
O(3)-C(1)	1.235(8)	O(16)-C(27)	1.458(7)
O(4)-C(1)	1.275(8)	O(16)-C(30)	1.360(8)
O(5)-C(16)	1.215(7)	O(17)-C(26)	1.425(8)
O(6)-C(13)	1.346(8)	O(18)-C(28)	1.322(8)
O(7)-C(15)	1.444(7)	O(19)-C(25)	1.420(8)
O(7)-C(16)	1.355(8)	C(25)-C(26)	1.523(9)
O(8)-C(14)	1.335(8)	C(25)-C(27)	1.524(8)
O(9)-C(17)	1.409(8)	C(27)-C(28)	1.496(9)
O(10)-C(18)	1.401(11)	C(28)-C(29)	1.362(8)
O(11)-C(18)	1.281(13)	C(29)-C(30)	1.440(9)
O(22)-C(34)	1.314(8)	O(20)-C(31)	1.430(8)
O(23)-C(35)	1.353(7)	O(21)-C(32)	1.417(8)
C(32)-C(33)	1.521(8)	O(24)-C(36)	1.218(7)
C(33)-C(34)	1.501(9)	O(25)-C(33)	1.460(7)
C(34)-C(35)	1.357(8)	O(25)-C(36)	1.363(7)
C(35)-C(36)	1.440(9)	C(31)-C(32)	1.514(9)
C(23)-N(3)-C(19)	122.6(6)	C(10)-C(9)-C(12)	119.8(6)
N(3)-C(19)-C(20)	119.3(6)	C(9)-C(10)-C(11)	118.8(6)
C(21)-C(20)-C(19)	119.7(6)	N(2)-C(11)-C(10)	119.6(6)
C(20)-C(21)-C(22)	119.2(6)	O(12)-C(12)-C(9)	115.6(5)
C(20)-C(21)-C(24)	121.4(5)	O(13)-C(12)-O(12)	126.0(6)
C(22)-C(21)-C(24)	119.4(5)	O(13)-C(12)-C(9)	118.3(6)
C(23)-C(22)-C(21)	119.3(6)	C(30)-O(16)-C(27)	108.4(5)

N(3)-C(23)-C(22)	119.9(6)	O(19)-C(25)-C(26)	107.8(5)
O(1)-C(24)-O(2)	125.6(6)	O(19)-C(25)-C(27)	110.3(5)
O(1)-C(24)-C(21)	118.4(5)	C(26)-C(25)-C(27)	113.1(5)
O(2)-C(24)-C(21)	116.0(5)	O(17)-C(26)-C(25)	112.2(5)
C(4)-N(1)-C(5)	122.2(6)	O(16)-C(27)-C(25)	110.4(5)
O(3)-C(1)-O(4)	126.2(6)	O(16)-C(27)-C(28)	104.0(5)
O(3)-C(1)-C(2)	117.9(6)	C(28)-C(27)-C(25)	112.7(5)
O(4)-C(1)-C(2)	115.9(5)	O(18)-C(28)-C(27)	118.1(5)
C(3)-C(2)-C(1)	119.1(5)	O(18)-C(28)-C(29)	132.4(6)
C(3)-C(2)-C(6)	120.1(6)	C(29)-C(28)-C(27)	109.5(6)
C(6)-C(2)-C(1)	120.7(6)	O(14)-C(29)-C(28)	134.7(6)
C(2)-C(3)-C(4)	118.9(6)	O(14)-C(29)-C(30)	118.2(5)
N(1)-C(4)-C(3)	120.2(6)	C(28)-C(29)-C(30)	107.1(6)
N(1)-C(5)-C(6)	120.3(6)	O(15)-C(30)-O(16)	120.5(6)
C(5)-C(6)-C(2)	118.3(6)	O(15)-C(30)-C(29)	128.5(6)
C(16)-O(7)-C(15)	108.9(5)	O(16)-C(30)-C(29)	111.0(5)
O(6)-C(13)-C(14)	134.2(6)	C(36)-O(25)-C(33)	108.4(5)
O(6)-C(13)-C(16)	118.5(5)	O(20)-C(31)-C(32)	111.4(5)
C(14)-C(13)-C(16)	107.4(6)	O(21)-C(32)-C(31)	106.5(5)
O(8)-C(14)-C(13)	133.7(6)	O(21)-C(32)-C(33)	111.2(5)
O(8)-C(14)-C(15)	116.6(5)	C(31)-C(32)-C(33)	113.2(5)
C(13)-C(14)-C(15)	109.5(5)	O(25)-C(33)-C(32)	112.2(5)
O(7)-C(15)-C(14)	104.1(5)	O(25)-C(33)-C(34)	104.1(4)
O(7)-C(15)-C(17)	110.6(5)	C(34)-C(33)-C(32)	112.1(5)
C(14)-C(15)-C(17)	116.0(6)	O(22)-C(34)-C(33)	116.9(5)
O(5)-C(16)-O(7)	121.3(6)	O(22)-C(34)-C(35)	133.9(6)
O(5)-C(16)-C(13)	128.6(6)	C(35)-C(34)-C(33)	109.1(5)
O(7)-C(16)-C(13)	110.1(5)	O(23)-C(35)-C(34)	134.0(6)
O(9)-C(17)-C(15)	110.4(5)	O(23)-C(35)-C(36)	118.2(5)
O(9)-C(17)-C(18)	110.7(6)	C(34)-C(35)-C(36)	107.8(5)
C(18)-C(17)-C(15)	110.3(6)	O(24)-C(36)-O(25)	120.1(6)
O(10)-C(18)-C(17)	118.7(8)	O(24)-C(36)-C(35)	129.3(6)
O(11)-C(18)-C(17)	116.0(7)	O(25)-C(36)-C(35)	110.6(5)
C(9)-C(8)-C(7)	118.9(6)	C(11)-N(2)-C(7)	123.0(6)
C(8)-C(9)-C(10)	120.2(6)	N(2)-C(7)-C(8)	119.5(6)
C(8)-C(9)-C(12)	119.9(6)		

Table S2. Hydrogen bond length (Å) and bond angle (°).

Hydrogen bond	D…A	D—H…A	Hydrogen bond	D…A	D—H…A
N(1)—H(1)…O(24A)	2.710(7)	173	O(8)—H(8)…O(12B)	2.547(7)	147
N(2)—H(2)…O(15B)	2.705(7)	163	O(9)—H(9)…O(11B)	2.591(11)	138
N(3)—H(3)…O(5B)	2.766(8)	168	O(14)—H(14)…O(3C)	2.560(8)	159
O(6)—H(6)…O(13B)	2.647(7)	159	O(17)—H(17A)…O(18D)	2.759(7)	159
O(8)—H(8)…O(12B)	2.547(7)	147	O(18)—H(18)…O(4C)	2.459(7)	152
O(9)—H(9)…O(11B)	2.591(11)	138	O(19)—H(19)…O(17B)	2.700(7)	172
O(14)—H(14)…O(3C)	2.560(8)	159	O(20)—H(20)…O(12)	2.788(7)	153(11)
O(17)—H(17A)…O(18D)	2.759(7)	159	O(21)—H(21)…O(20B)	2.728(7)	173(7)
O(18)—H(18)…O(4C)	2.459(7)	152	O(22)—H(22)…O(2E)	2.524(7)	156
N(1)—H(1)…O(24A)	2.710(7)	173	O(23)—H(23)…O(1E)	2.622(7)	158
N(2)—H(2)…O(15B)	2.705(7)	163	O(6)—H(6)…O(13B)	2.647(7)	159
N(3)—H(3)…O(5B)	2.766(8)	168			

A: -1/2+x,-1/2+y,z; B: x,-1+y,z; C: 1-x,-1+y,1-z; D: 3/2-x,1/2+y,1-z; E: 1-x,-1+y,-z.
